# The functions of a reservoir offset voltage applied to physically defined p-channel Si quantum dots

**DOI:** 10.1038/s41598-022-14669-x

**Published:** 2022-06-21

**Authors:** Shimpei Nishiyama, Kimihiko Kato, Mizuki Kobayashi, Raisei Mizokuchi, Takahiro Mori, Tetsuo Kodera

**Affiliations:** 1 Department of Electrical and Electronic Engineering, Tokyo Institute of Technology, Meguro, Tokyo, 152-8552 Japan; 2 Device Technology Research Institute (D-Tech), National Institute of Advanced Industrial Science and Technology (AIST) Tsukuba, Ibaraki, 305-8568 Japan

**Keywords:** Electrical and electronic engineering, Quantum dots, Electronic and spintronic devices, Qubits, Electronic devices

## Abstract

We propose and define a reservoir offset voltage as a voltage commonly applied to both reservoirs of a quantum dot and study the functions in p-channel Si quantum dots. By the reservoir offset voltage, the electrochemical potential of the quantum dot can be modulated. In addition, when quantum dots in different channels are capacitively coupled, the reservoir offset voltage of one of the QDs can work as a gate voltage for the others. Our results show that the technique will lead to reduction of the number of gate electrodes, which is advantageous for future qubit integration.

## Introduction

A spin in a Si quantum dot (QD) is an attractive system for implementing quantum information processing because it has a small qubit size and relatively long coherence times which come from a low abundance of nuclear spins in natural Si^1–3^. In addition, the mature conventional complementary metal–oxide–semiconductor (CMOS) fabrication process can be applied for Si QD fabrication, which should be advantageous for large-scale qubit integration^4–6^. Recently, several research groups have been studying various Si QD structures toward the integration of Si spin qubits^7–12^. One issue for the integration is that increasing the number of gate electrodes per qubit leads to increasing the number of wires from room temperature, which conflicts the limitation of space for wiring at cryogenic temperatures. In addition, characteristic variation among qubits will also become a problem for the integration^13,14^. To tackle these problems, we proposed and utilized a reservoir offset voltage which is a voltage commonly applied to both reservoirs of a QD. In the study of transistors, this voltage is understood to work as a relative gate voltage. Here, we apply the technique to QDs and thereby demonstrate the use of the reservoirs of a QD as gate electrode. We believe that the technique leads to a reduction of the number of gates without complicating structures.

Our group has been fabricating physically defined Si QD devices^15–21^, which enable the potential modulation methods using the reservoir offset voltage by taking advantages of the strong, sharp quantum confinements. In the present study, we confirm the operating principle of the reservoir offset voltage by simulation and applied the technique to p-channel QD devices. In p-channel Si QDs, hole spin manipulation is possible only with AC electric fields thanks to strong spin–orbit couplings of holes, which is advantageous for future qubit integration^22–25^. At first, we perform a band structure simulation to investigate the detailed mechanism of the reservoir offset voltage technique. Next, we experimentally demonstrate potential energy tuning in QDs by the reservoir offset voltage at 4.2 K and confirm that the operating principle shown in the simulation can explain the experimental results.

## Results

### Device and measurement setup

**Figure 1:**
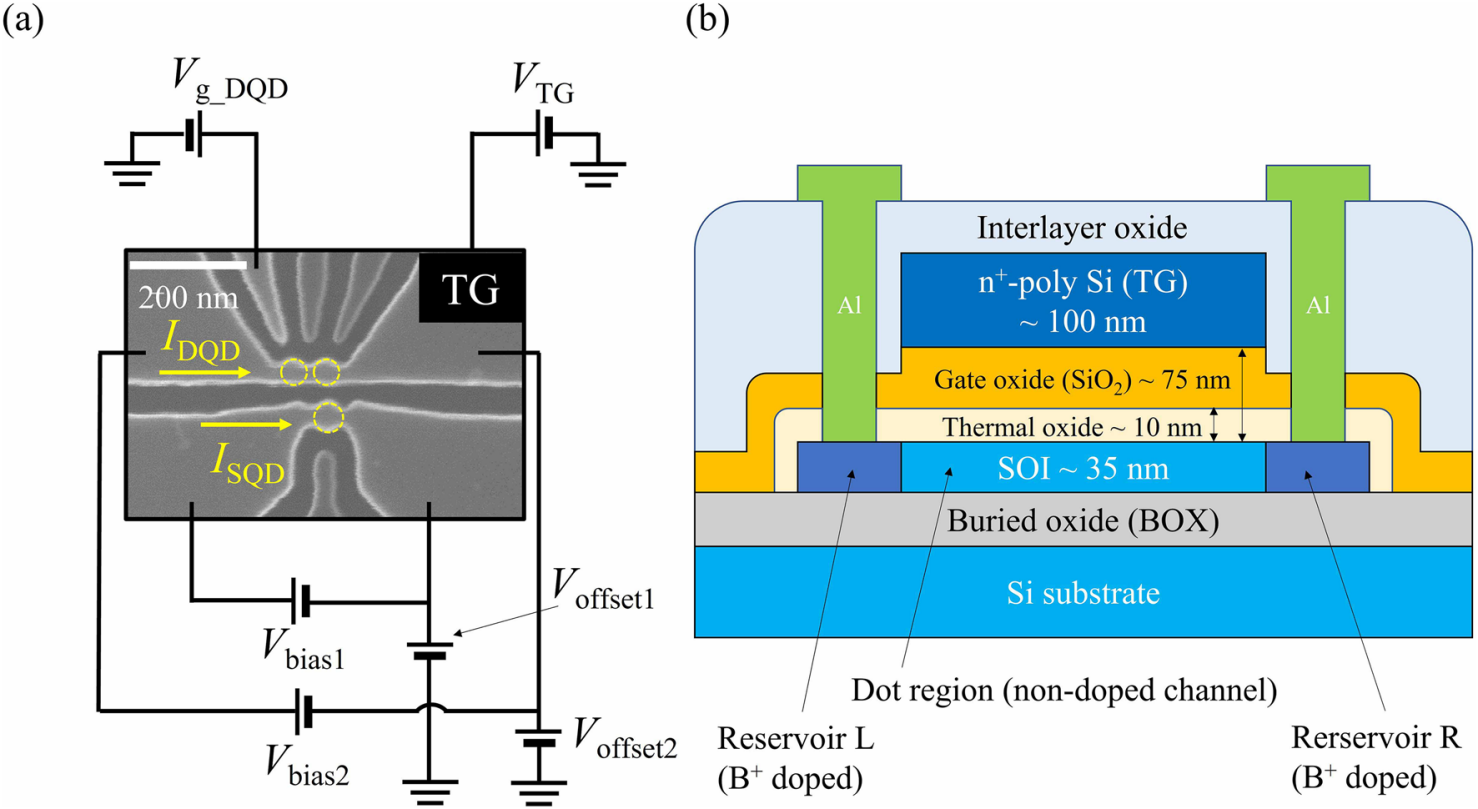
(**a**) A scanning electron micrograph (SEM) of the physically defined QD device. QDs in this device are indicated by yellow dashed circles. A single QD (SQD) located next to a double QD (DQD) is usually used as a charge sensor to observe the charge states in the DQD. (**b**) A cross-sectional schematic of the device structure. A poly-Si top gate on a 75 nm-thick SiO_2_ layer covers the QD region. Boron ions are implanted at the outside region where the top gate does not cover silicon on insulator (shown by dark blue region).

Figure 1a shows the experimental setup with a scanning electron microscope (SEM) image of a physically defined p-channel Si QD system. The fabrication process is in line with our previous studies^16–21^. At first, a 40 nm-thick silicon-on-insulator (SOI) substrate is etched to form single QD (SQD), double QD (DQD), and side gates (SGs) by reactive ion etching technique after electron beam lithography. Next, following thermal passivation of the Si surface, a 75 nm-thick SiO_2_ layer and then a poly-Si top gate (TG) are deposited by low-pressure chemical vapor deposition technique. Then, to create Ohmic contacts, boron ions are implanted into SOI layer except for the part of SOI which is covered with TG with the accelerating voltage of 22 keV and a doping density of $1.1 \times 10^{20} \;{\text{cm}}^{ - 3}$. Finally, forming gas annealing is performed to terminate dangling bonds at 420 °C for 3 h. The cross-sectional schematic of the QD device structure is shown in Fig. 1b. In our device structure, TG voltage accumulates holes in both SQD and DQD, and each SG voltage modifies the potential of the neighboring QD mainly. As described below, a reservoir offset voltage can be utilized to effectively tune the entire potential of a QD system (e.g. SQD or DQD system in this device). Therefore, the technique can be regarded to have a different function from the gates and potentially replaces TG and/or SG.

### Band structure simulation model

**Figure 2:**
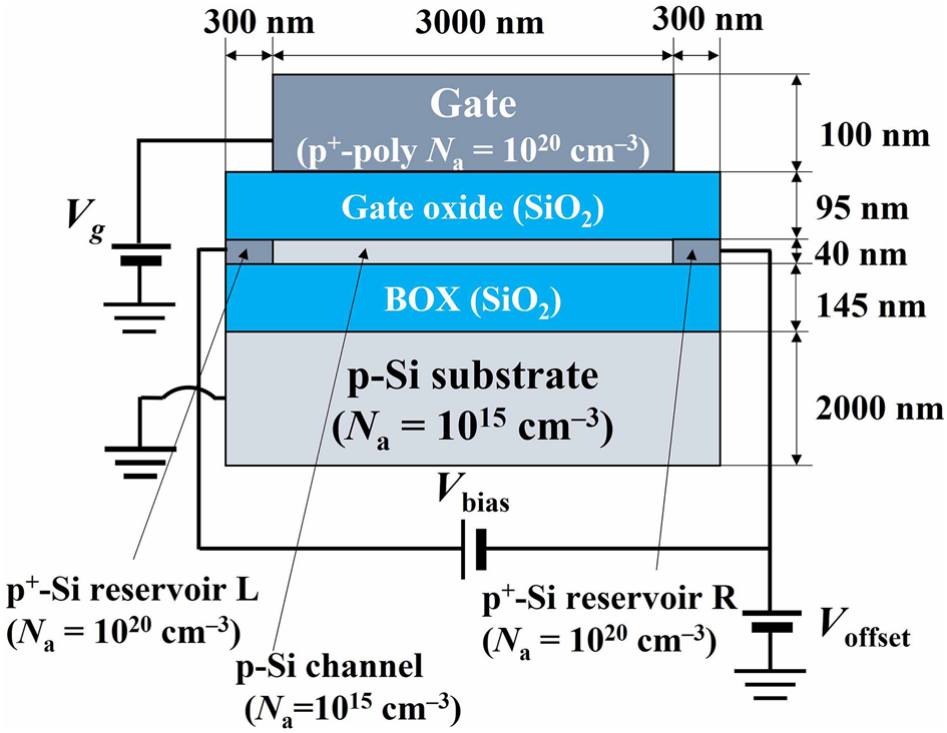
A simple MOS structure model used in device simulation to investigate the effect of the reservoir offset voltage on charge accumulation of Si channel qualitatively. This device is simulated at a temperature of 300 K, and the width of the channel is not defined for simplicity. The impurity concentration of each layer, indicated in the label, is chosen based on the actual device.

In order to study the mechanism of the reservoir offset voltage technique, we calculated energy band structures employing HyENEXSS™^26–28^. Here, we performed simulation using a simple p-channel MOS field effect transistor (MOSFET) model to study the changes in the energy bands and the number of holes induced in the reservoirs by the reservoir offset voltage (Fig. 2). As shown in Fig. 1b, the actual device structure except for QD is similar to a typical MOSFET structure, so that the change in the reservoir potential by the reservoir offset voltage qualitatively corresponds to that of the simulated MOSFET model. To confirm the effect of the reservoir offset voltage on the reservoirs, the lateral quantum confinement of the QD is not considered in this simulation. This means that the width of the Si channel is not considered for simplicity. The simulation temperature is chosen to be 300 K because the simulation at low temperatures has the difficulty in convergence^29–31^ due to the low intrinsic carrier density. Note that our target in the simulation is to grab the trend of the change in the energy band structures and the Fermi level by the reservoir offset voltage, so that the quantitative difference occurred by the different temperatures would not be critical for the conclusion. Even with such a simple simulation, the mechanism of the potential control by the reservoir offset voltage can be qualitatively understood.

### Simulation results for the reservoir offset voltage measurement

**Figure 3:**
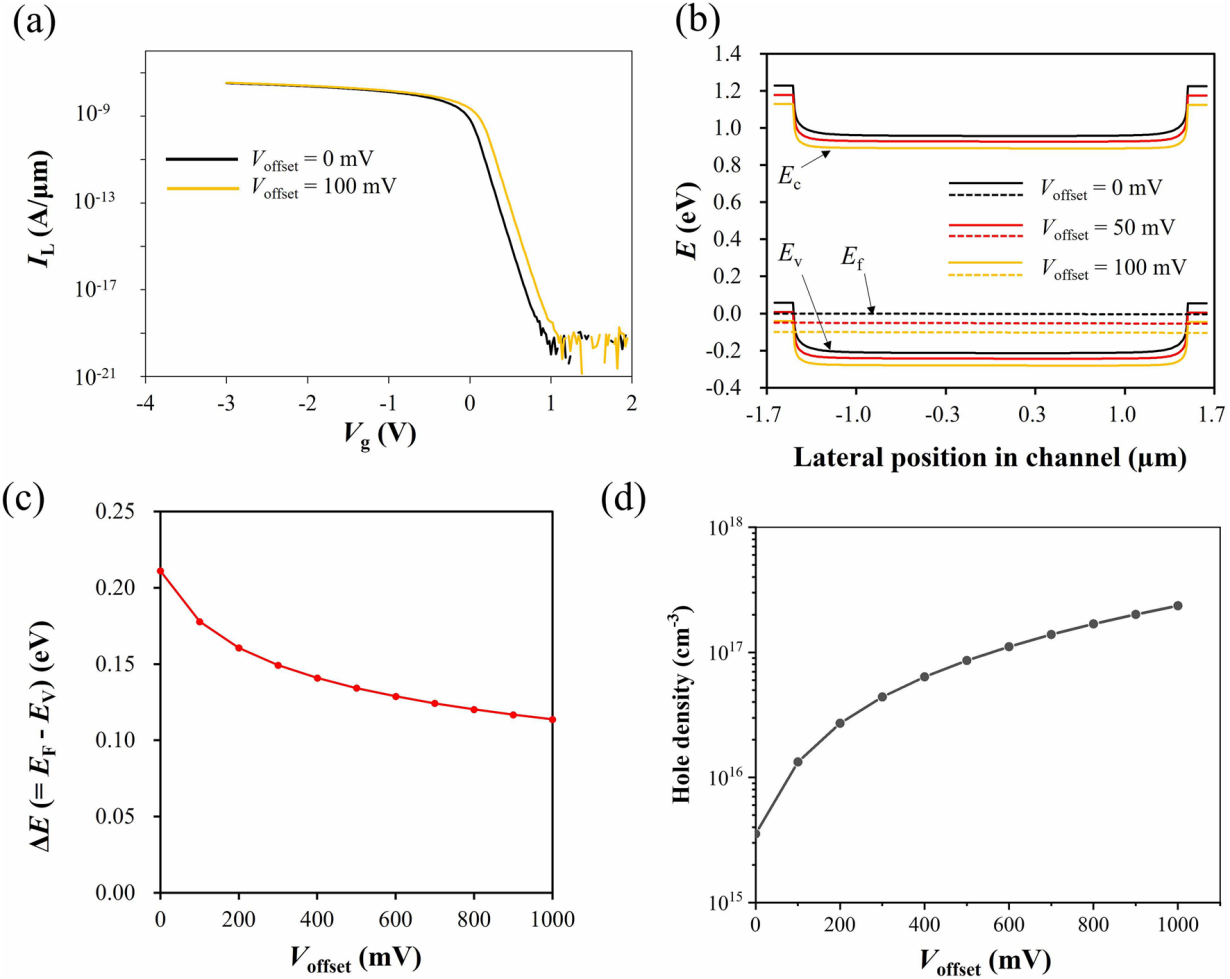
Simulation results. (**a**) Current at reservoir L, *I*_L_ of the device shown in Fig. 2 as a function of *V*_g_ when *V*_offset_ = 0 mV (black) or 100 mV (orange). At *V*_offset_ = 100 mV, the threshold voltage of the device shifts slightly in the positive direction. (**b**) Simulated energy band diagrams on the surface of Si channel in MOS structure model at 300 K. *V*_g_ = 0 V (threshold region), *V*_bias_ = 4 mV, and *V*_offset_ = 0 mV (black), 50 mV (red), and 100 mV (orange). All the valence band edge (*E*_V_), the conduction band edge (*E*_C_), and the Fermi level (*E*_F_) shift depending on *V*_offset_. (**c**) The reservoir offset voltage dependence of the difference between *E*_F_ and *E*_V_ ($\Delta E$) at *V*_g_ = 0 V and *V*_bias_ = 4 mV when the range of *V*_offset_ expanded to hundreds of millivolts. Since $\Delta E$ corresponds to the hole concentration induced in the Si layer, the reservoir offset voltage has a function to tune the number of induced holes in the reservoirs. (**d**) A simulated hole density on the top of the Si channel depending on the reservoir offset voltage *V*_offset_ at *V*_g_ = 0 V and 300 K.

Figure 3a shows the simulated *I*_L_–*V*_g_ characteristics with *V*_offset_ = 0 mV (black line) and 100 mV (orange line). We found that the threshold voltage shifts by about 100 mV. This is because the reservoir offset voltage causes a potential difference between the channel and the gate. In this case, the positive reservoir offset voltage is applied such that it accumulates holes. Typically, in MOS-based QD devices, Coulomb oscillations appear in the threshold region^32–34^. Therefore, the offset bias effect is confirmed for *V*_g_ fixed close to the threshold voltage in the calculation of the energy bands. The valence band edge *E*_V_, the conduction band edge *E*_C_, and the Fermi level *E*_F_ at Si surface along the channel direction at *V*_g_ = 0 V are calculated with changing *V*_offset_ from 0 to 100 mV as a simulation parameter. The results obtained for *V*_offset_ = 0 mV (black), 50 mV (red), and 100 mV (orange) are shown in Fig. 3b. The energy bands shift to the negative direction along *y* axis by the positive reservoir offset voltage; in addition, the energy difference ∆*E* between *E*_V_ and *E*_F_ also changes at the same time. The number of induced holes at the Si/SiO_2_ interface depends on ∆*E*. Figure 3c shows ∆*E* at the center of the channel on Si/SiO_2_ interface as a function of the reservoir offset voltage *V*_offset_ for *V*_g_ = 0 V and *V*_bias_ = 4 mV. It turned out that the increase of *V*_offset_ reduces ∆*E* and thus increases the number of holes induced in reservoirs (Fig. 3d). Note that a moderate hole accumulation by *V*_g_ is needed to observe the band edge difference by *V*_offset_ in the simulations; ∆*E* ~ 0 for a stronger accumulation, and no band shift in the channel for a weaker hole accumulation. From the simulation, we confirmed that the reservoir offset voltage can control the Fermi level and the number of holes in the channel due to the adjustment of ∆*E*.

### Potential tuning by the reservoir offset voltage measurements

**Figure 4:**
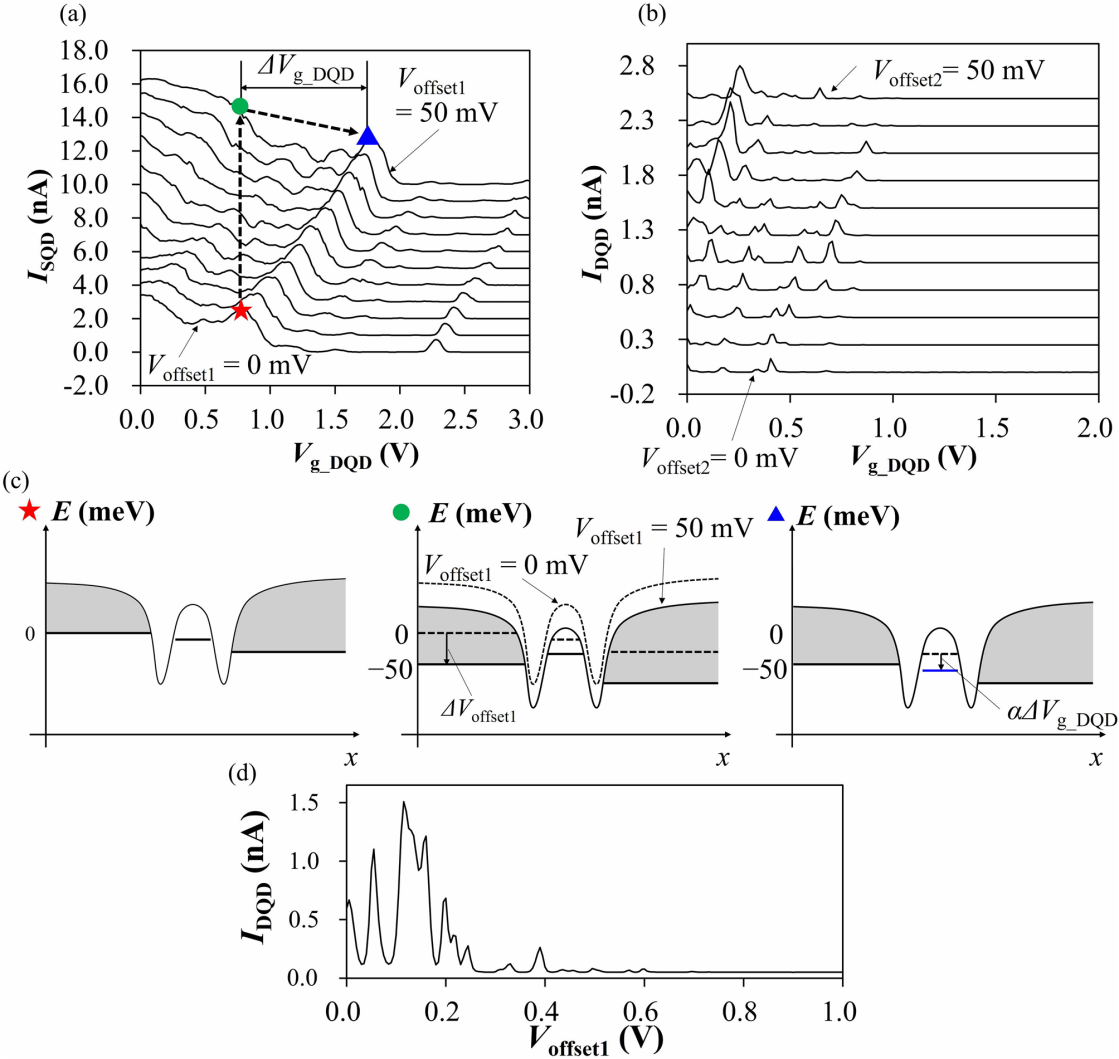
Measurement results. (**a**,**b**) Measured current through (**a**) SQD ((**b**) DQD) as a function of *V*_g_DQD_ at different reservoir offset voltages, *V*_offset1_ (*V*_offset2_) between 0 mV (bottom) and 50 mV (top). *V*_TG_ = − 1.7 V, *V*_bias1_ = 4 mV in (**a**), *V*_bias2_ = 1 mV in (**b**) at a temperature of 4.2 K. Each trace is given an offset of 1.0 nA in (**a**) and 0.25 nA in (b) for clarity, respectively. The DQD current in (**b**) shows an SQD-like behavior because two QDs of the DQD are strongly coupled to each other. These position shifts correspond to potential modulation in the QD generated by the reservoir offset voltage as shown in the diagrams in (**c**). (**c**) Energy diagrams for red-star (left), green-circle (center) and blue-triangle (right) conditions in (**a**), respectively. *x* axis indicates the lateral position of channel including QD. (**d**) Current through the DQD as a function of *V*_offset1_ applied to the SQD. *V*_TG_ = − 1.7 V and *V*_bias1_ = 1.0 mV are fixed in this measurement.

Next, we experimentally demonstrated the potential modulation in p-channel QD systems by the reservoir offset voltage at a temperature of 4.2 K. Figure 4a,b show Coulomb peaks through an SQD and a DQD at *V*_TG_ =  − 1.7 V as a function of a voltage from the left SG close to left dot, *V*_g_DQD_, respectively. Positive reservoir offset voltages, *V*_offset1_ (*V*_offset2_), are applied to the SQD (DQD), and thereby, peak positions of the SQD (DQD) characteristic are shifted to positive *V*_g_DQD_ direction as the reservoir offset voltage increases. Considering from the simulation results in Fig. 3b, the reservoir offset voltage affects both discrete energies of the QD and the Fermi levels of the reservoirs. Here, we consider the condition that an energy level of the SQD is in the bias window at *V*_offset1_ of 0 mV (red star in Fig. 4c).

When *V*_offset1_ of 50 mV is applied, changing from the condition at the red star, a positive reservoir offset voltage shifts the Fermi levels into the direction that the hole number in the QD increases, which plays the same role as a negative gate voltage. On the other hand, the positive reservoir offset voltage accumulates holes in reservoirs and works like a positive gate voltage for the QD as well, through Coulomb repulsion (green circle in Fig. 4c).

In order to know how much energy shift occurs by reservoir offset voltage, a positive SG voltage is applied to compensate the energy shift. When the SG voltage compensation and the reservoir offset voltage are applied, the same Coulomb peak as without SG voltage compensation and reservoir offset voltage appears (blue triangle in Fig. 4c). Therefore, the potential shift of the SQD by the reservoir offset voltage, ${\Delta E}_{{{\text{QD}}}}$, can be roughly estimated from the relation, ${\Delta E}_{{{\text{QD}}}} = \upalpha {\Delta V}_{{{{\text{g}_\text{DQD}}}}}$, where $\upalpha$ is the coupling strength between the SG and the SQD, and ${\Delta V}_{{{{\text{g}_\text{DQD}}}}}$ is the SG voltage difference between two Coulomb peaks for the green-circle and blue-triangle conditions. For *V*_offset1_ of 50 mV, ${\Delta E}_{{{\text{QD}}}}$ is estimated to be $\sim 10 \;{\text{meV}}$, where $\upalpha \sim {0}{\text{.01}}$ eV/V^18^ and ${\Delta V}_{{{{\text{g}_\text{DQD}}}}} {\text{ = 950 mV}}$. The shift is smaller than that of the Fermi level shift $eV_{{{\text{offset}}}} = {\text{50 meV}}$, which is attributed to the Coulomb coupling between the reservoirs and the SQD as mentioned above. From the energy difference between $eV_{{{\text{offset}}}}$ and ${\Delta }E_{{{\text{QD}}}}$, $\Delta E_{{{\text{r}} - {\text{QD}}}} = eV_{{{\text{offset}}}} - \Delta E_{{{\text{QD}}}} \sim 40\;{\text{meV}}$, the coupling strength between the reservoir and the SQD, $\upalpha_{{{\text{r}} - {\text{QD}}}} = \Delta E_{{{\text{r}} - {\text{QD}}}} /V_{{{\text{offset}}}}$ is estimated to be 0.8 eV/V, which is compatible to that calculated from the parameters for the same type of the QD ($\upalpha_{{{\text{r}} - {\text{QD}}}}  = 0{\text{.7}}$ eV/V)^18^. This is the principle of the potential modulation function of the reservoir offset voltage. The potential modulation function will facilitate the realization of the single-hole limit; it can reduce the number of holes while lowering the tunnel barrier heights. On the other hand, a side gate voltage can also reduce the number of holes but increases the barrier heights. This difference comes from the sign of the applied voltages and indicates an advantage of the reservoir offset voltage technique.

### Gating function of the reservoir offset voltage

To observe the other function of the reservoir offset voltage, gating function, we performed another measurement. In the measurement, we applied a reservoir offset voltage to an SQD and monitored the current flowing through the nearby DQD. Here, we expand the operating range of the reservoir offset voltage to hundreds of millivolts for visibility of the gating function. Figure 4d shows Coulomb peaks of DQD as a function of *V*_offset1_, and they disappear as *V*_offset1_ increases, in analogy with SG voltage dependence. In our device, the DQD and SQD are physically isolated from each other but still electrostatically coupled. Therefore, as pointed out in Fig. 3c, *V*_offset1_ is considered to induce the gating effect by hole accumulation in the reservoirs of the SQD. From these results, we confirmed that not only SGs but also a QD can be used to tune the potential of another QD by applying the reservoir offset voltage, possibly leading to further reduction of the number of gates.

## Conclusion

In conclusion, we study the functions of the reservoir offset voltages from simulation and experiments for physically defined p-channel Si QDs. We demonstrate that the reservoir offset voltages control the potentials of an SQD and a DQD relatively to the Fermi level, respectively. In addition, we observe that the reservoir offset voltage to the SQD works as a gate voltage for the adjacent DQD through Coulomb repulsion due to accumulated holes in the SQD reservoirs. From these results, we conclude that the reservoir offset voltage can lead to reduction of gate electrodes and/or flexibility of control in physically defined Si QD devices. We believe that this technique is advantageous also for other QDs based on different semiconductors, with strong isolation such as physically defined and nanowire-based QDs^35,36^.

## Data Availability

The datasets generated during and/or analysed during the current study are available from the corresponding author on reasonable request.

## References

[1] Takeda K, Noiri A, Yoneda J, Nakajima T, Tarucha S (2020). Resonantly driven singlet-triplet spin qubit in silicon. Phys. Rev. Lett..

[2] de Sousa R, Das Sarma S (2003). Theory of nuclear-induced spectral diffusion: Spin decoherence of phosphorus donors in Si and GaAs quantum dots. Phys. Rev. B.

[3] Huang W (2019). Fidelity benchmarks for two-qubit gates in silicon. Nature.

[4] Itoh KM, Watanabe H (2014). Isotope engineering of silicon and diamond for quantum computing and sensing applications. MRS Commun..

[5] Takeda K (2016). A fault-tolerant addressable spin qubit in a natural silicon quantum dot. Sci. Adv..

[6] Maurand R (2016). A CMOS silicon spin qubit. Nat. Commun..

[7] Veldhorst M (2014). An addressable quantum dot qubit with fault-tolerant control-fidelity. Nat. Nanotechnol..

[8] Veldhorst M (2017). Silicon CMOS architecture for a spin-based quantum computer. Nat. Commun..

[9] Kawakami E (2014). Electrical control of a long-lived spin qubit in a Si/SiGe quantum dot. Nat. Nanotechnol..

[10] Ahmed I (2018). Radio-frequency capacitive gate-based sensing. Phys. Rev. Appl..

[11] Crippa A (2019). Gate-reflectometry dispersive readout and coherent control of a spin qubit in silicon. Nat. Commun..

[12] Yoneda J (2020). Quantum non-demolition readout of an electron spin in silicon. Nat. Commun..

[13] Mizuno T, Okamura J, Toriumi A (1994). Experimental study of threshold voltage fluctuation due to statistical variation of channel dopant number in MOSFET's. IEEE Trans. Electron. Devices.

[14] Nishinohara K, Shigyo N, Wada T (1992). Effects of microscopic fluctuations in dopant distributions on MOSFET threshold voltage. IEEE Trans. Electron. Devices.

[15] Yamahata G (2012). Magnetic field dependence of Pauli spin blockade: A window into the sources of spin relaxation in silicon quantum dots. Phys. Rev. B.

[16] Horibe K, Kodera T, Oda S (2015). Lithographically defined few-electron silicon quantum dots based on a silicon-on-insulator substrate. Appl. Phys. Lett..

[17] Horibe K, Kodera T, Oda S (2015). Back-action-induced excitation of electrons in a silicon quantum dot with a single-electron transistor charge sensor. Appl. Phys. Lett..

[18] Yamaoka Y, Iwasaki K, Oda S, Kodera T (2017). Charge sensing and spin-related transport property of p-channel silicon quantum dots. Jpn. J. Appl. Phys..

[19] Mizokuchi R, Oda S, Kodera T (2019). Physically defined triple quantum dot systems in silicon on insulator. Appl. Phys. Lett..

[20] Wei H, Mizoguchi S, Mizokuchi R, Kodera T (2020). Estimation of hole spin g-factors in p-channel silicon single and double quantum dots towards spin manipulation. Jpn. J. Appl. Phys..

[21] Kambara T, Kodera T, Arakawa Y, Oda S (2013). Dual function of single electron transistor coupled with double quantum dot: Gating and charge sensing. Jpn. J. Appl. Phys..

[22] Pribiag VS (2013). Electrical control of single hole spins in nanowire quantum dots. Nat. Nanotechnol..

[23] Hu Y, Kuemmeth F, Lieber CM, Marcus CM (2012). Hole spin relaxation in Ge–Si core–shell nanowire qubits. Nat. Nanotechnol..

[24] Hung JT (2017). Spin blockade in hole quantum dots: Tuning exchange electrically and probing Zeeman interactions. Phys. Rev. B.

[25] Marcellina A (2017). Spin-orbit interactions in inversion-asymmetric two-dimensional hole systems: A variational analysis. Phys. Rev. B.

[26] Kotani, N. *TCAD in Selete. Proc. Int. Conf. SISPAD* 3 (1998).

[27] Wada, T. *et al.* ENEXSS a 3-dimensional TCAD system. *Proc. Ext. Abstr. 53rd Spring Meeting Jpn. Soc. Appl. Phys.* 7 (2006).

[28] Nakamura M (2008). Current status and subjects on practical 3D TCAD for next generation. Jpn. Soc. Appl. Phys..

[29] Selberherr S (1989). MOS device modeling at 77 K. IEEE Trans. Electron Devices.

[30] Jaeger RC, Gaensslen FH (1980). Simulation of impurity freezeout through numerical solution of Poisson's equation with application to MOS device behavior. IEEE Trans. Electron Devices.

[31] Beckers A (2020). Physical model of low-temperature to cryogenic threshold voltage in MOSFETs. IEEE J. Electron Devices Soc..

[32] Voisin B (2014). Few-electron edge-state quantum dots in a silicon nanowire field-effect transistor. Nano Lett..

[33] Ishikuro H, Hiramoto T (1997). Quantum mechanical effects in the silicon quantum dot in a single-electron transistor. Appl. Phys. Lett..

[34] Angus SJ, Ferguson AJ, Dzurak AS, Clark RG (2007). Gate-defined quantum dots in intrinsic silicon. Nano Lett..

[35] Watzinger H, Kloeffel C, Vukusic L, Rossell MD, Sessi V, Kukucka J, Kirchschlager R, Lausecker E, Truhlar A, Glaser M, Rastelli A, Fuhrer A, Loss D, Katsaros G (2016). Heavy-hole states in germanium hut wires. Nano Lett..

[36] Brauns M, Ridderbos J, Li A, van der Wiel WG, Bakkers EPAM, Zwanenburg AF (2016). Highly tuneable hole quantum dots in Ge-Si core-shell nanowires. Appl. Phys. Lett..

